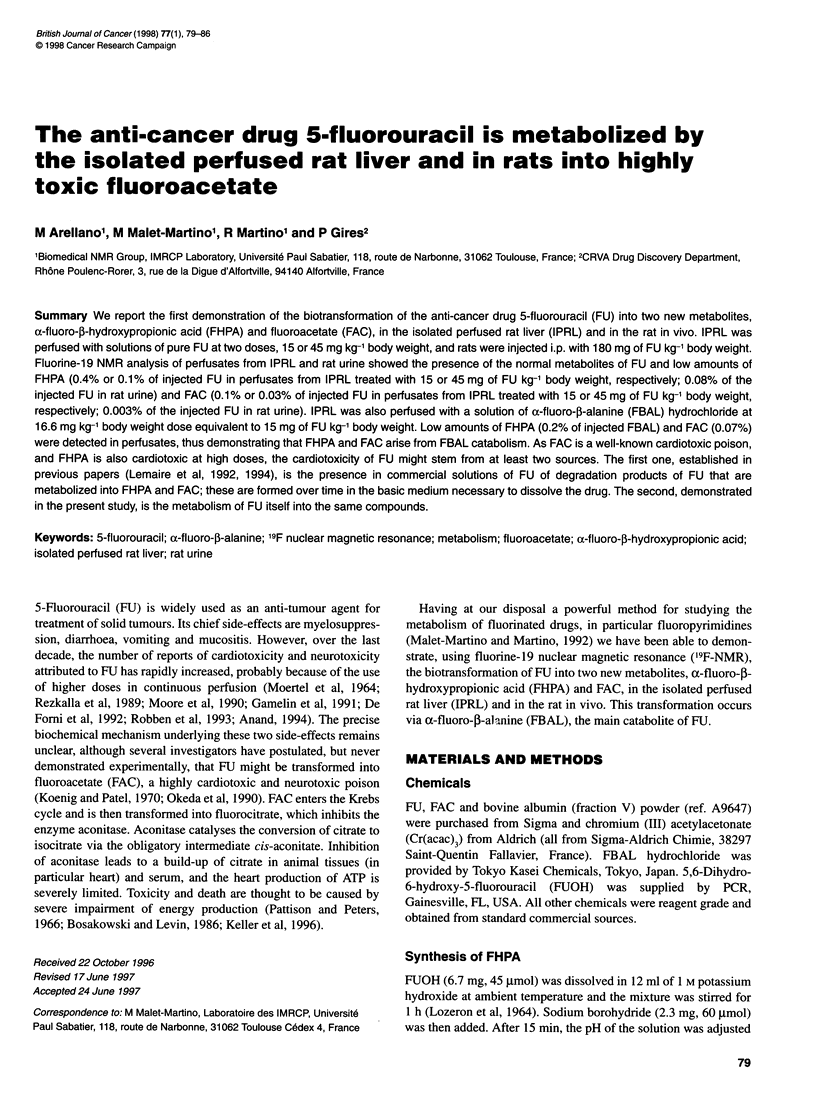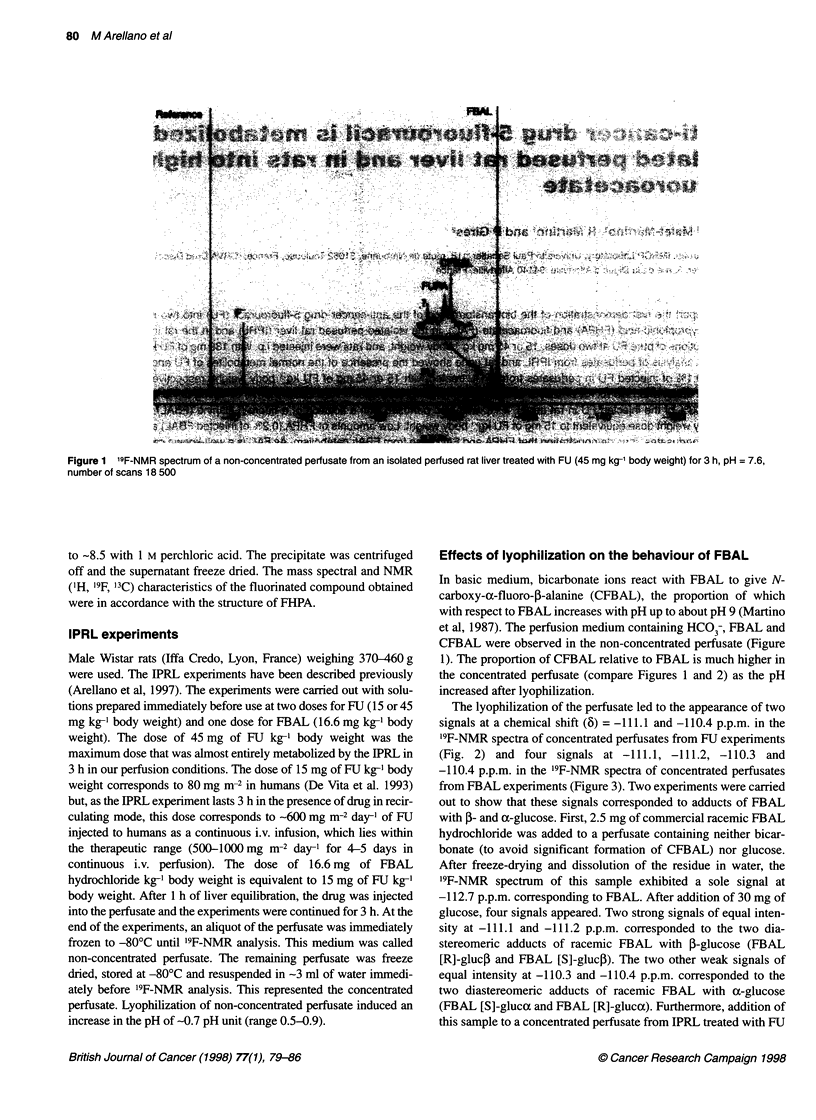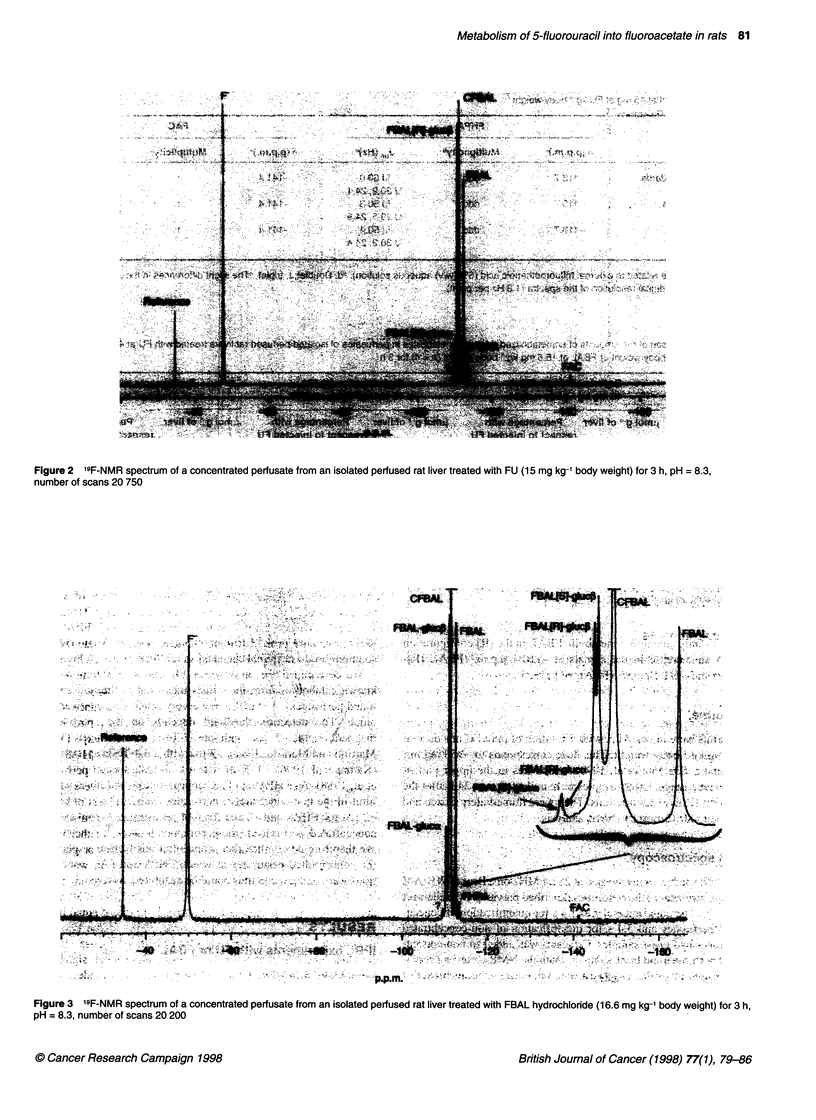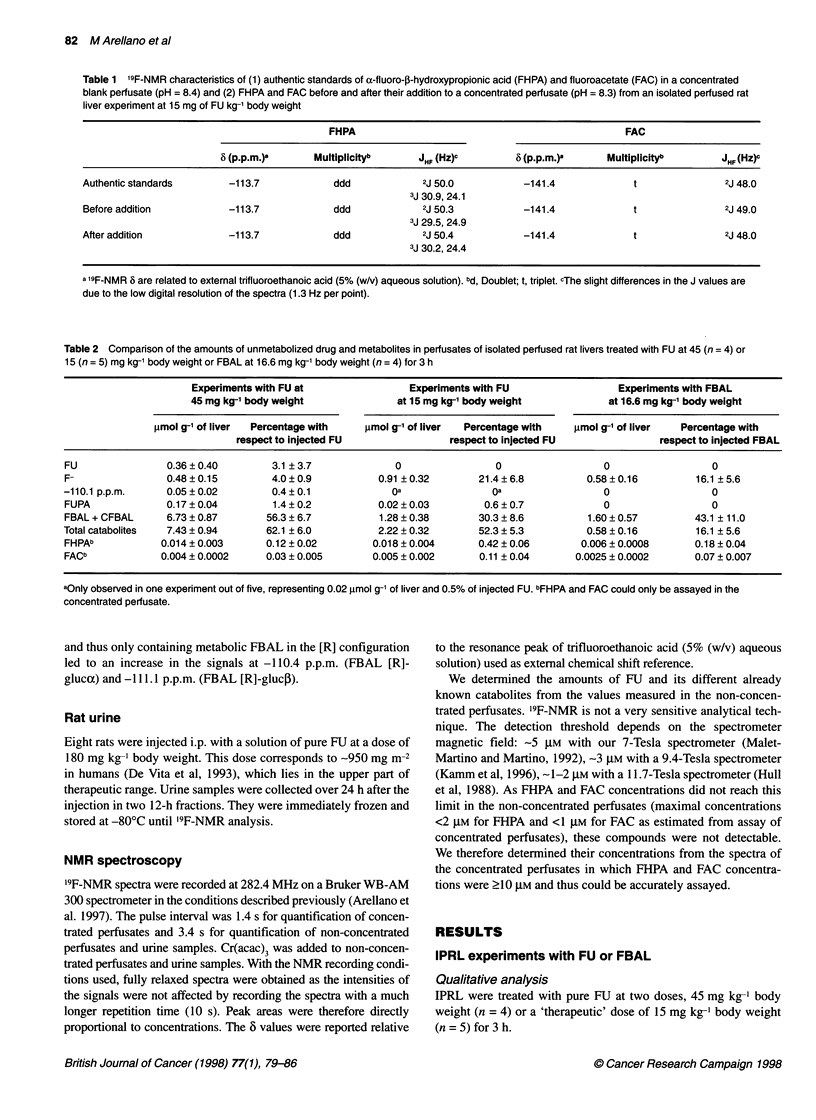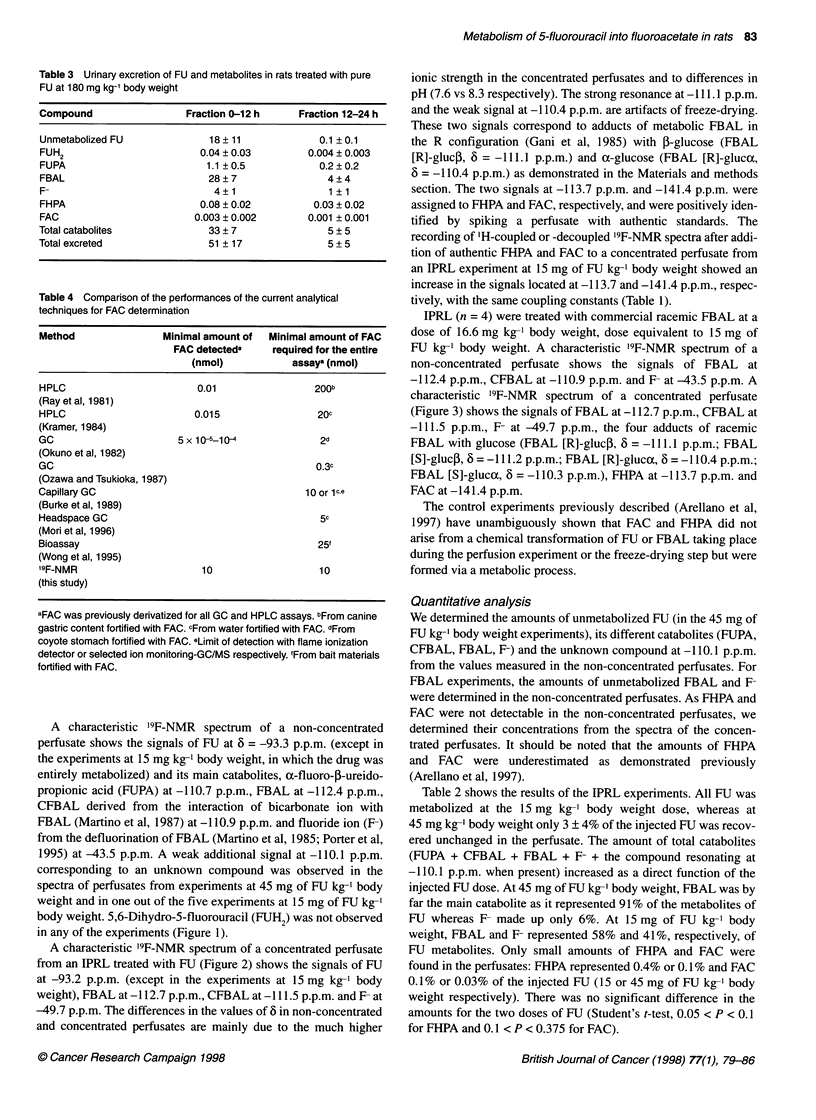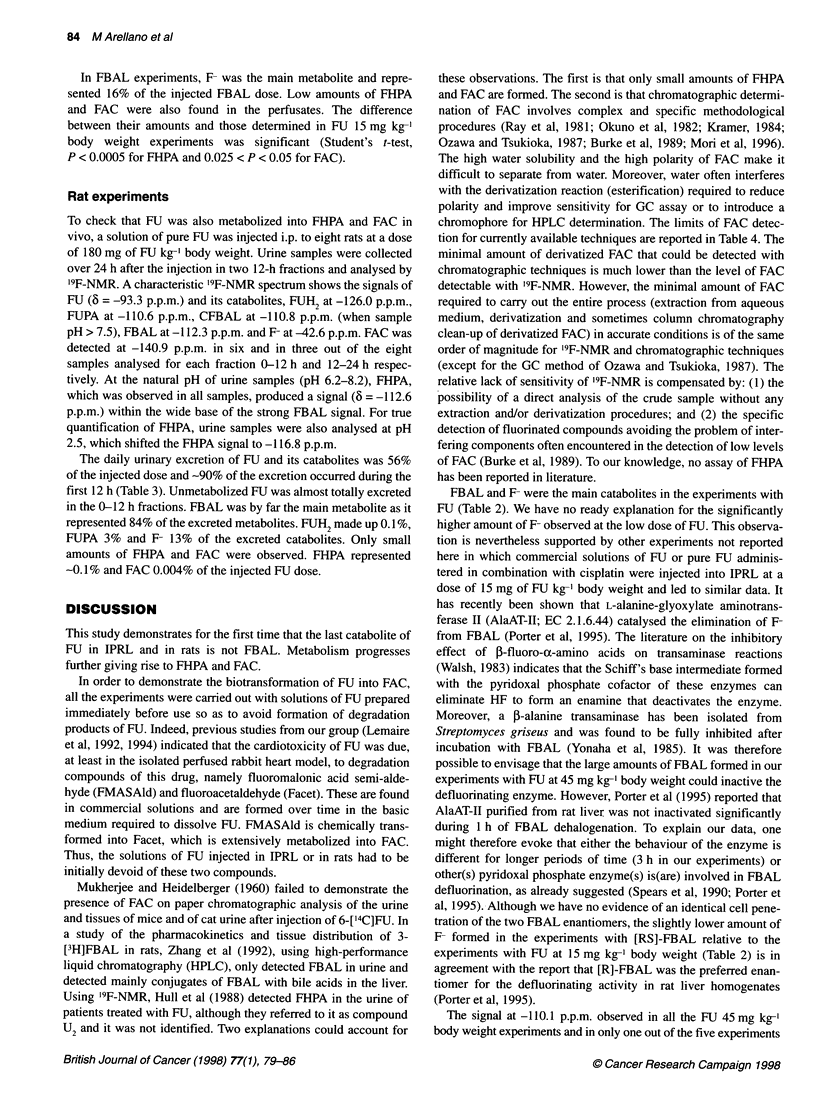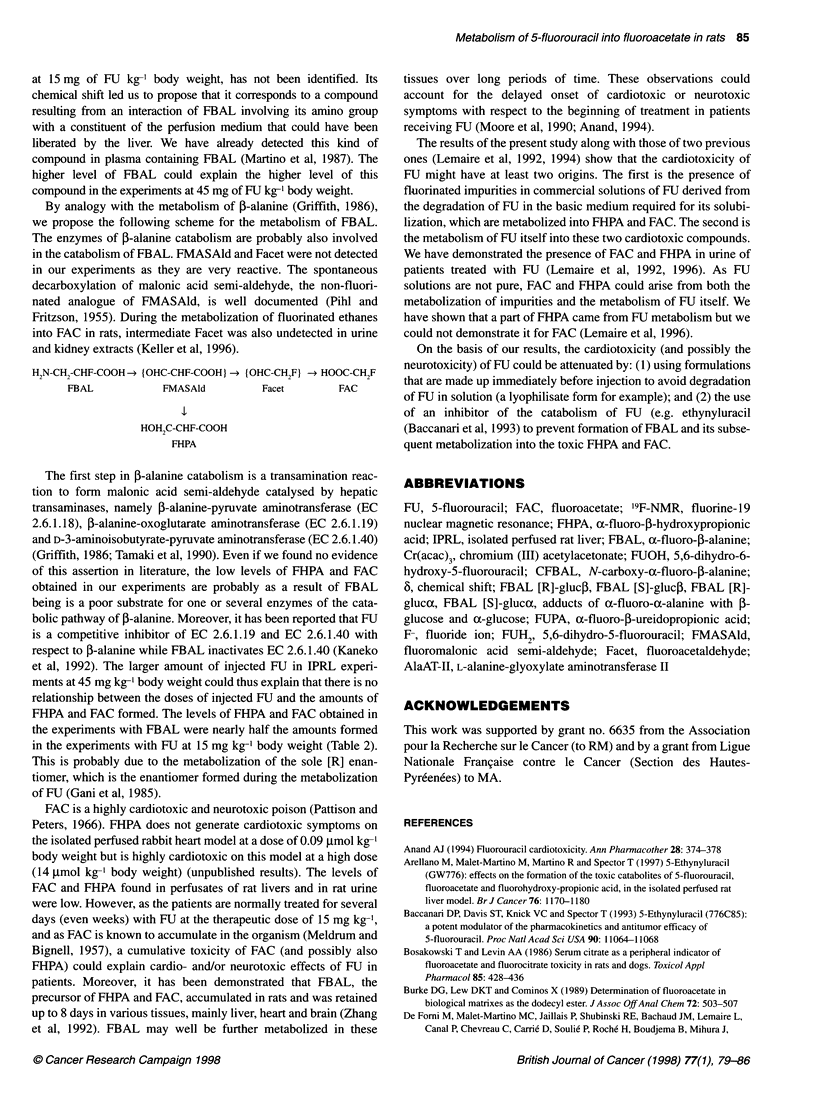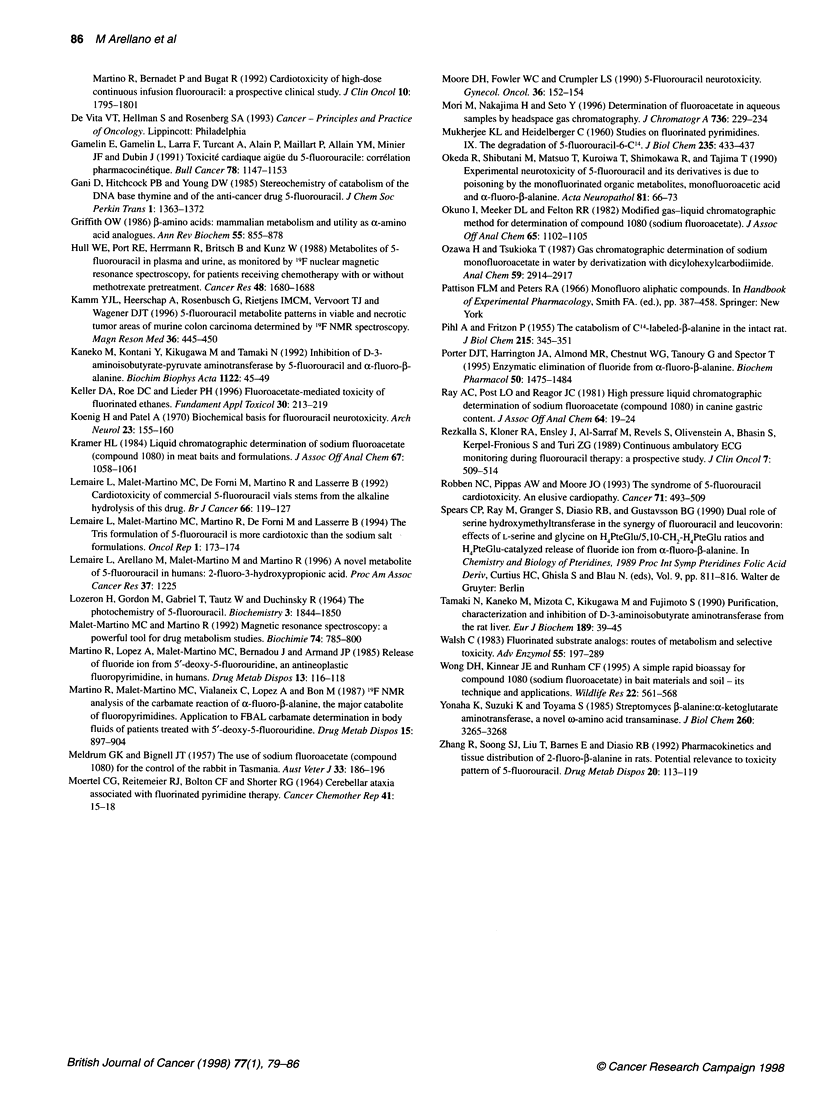# The anti-cancer drug 5-fluorouracil is metabolized by the isolated perfused rat liver and in rats into highly toxic fluoroacetate.

**DOI:** 10.1038/bjc.1998.12

**Published:** 1998

**Authors:** M. Arellano, M. Malet-Martino, R. Martino, P. Gires

**Affiliations:** Biomedical NMR Group, IMRCP Laboratory, UniversitÃ© Paul Sabatier, Toulouse, France.

## Abstract

We report the first demonstration of the biotransformation of the anti-cancer drug 5-fluorouracil (FU) into two new metabolites, alpha-fluoro-beta-hydroxypropionic acid (FHPA) and fluoroacetate (FAC), in the isolated perfused rat liver (IPRL) and in the rat in vivo. IPRL was perfused with solutions of pure FU at two doses, 15 or 45 mg kg(-1) body weight, and rats were injected i.p. with 180 mg of FU kg(-1) body weight. Fluorine-19 NMR analysis of perfusates from IPRL and rat urine showed the presence of the normal metabolites of FU and low amounts of FHPA (0.4% or 0.1% of injected FU in perfusates from IPRL treated with 15 or 45 mg of FU kg(-1) body weight, respectively; 0.08% of the injected FU in rat urine) and FAC (0.1% or 0.03% of injected FU in perfusates from IPRL treated with 15 or 45 mg of FU kg(-1) body weight, respectively; 0.003% of the injected FU in rat urine). IPRL was also perfused with a solution of alpha-fluoro-beta-alanine (FBAL) hydrochloride at 16.6 mg kg(-1) body weight dose equivalent to 15 mg of FU kg(-1) body weight. Low amounts of FHPA (0.2% of injected FBAL) and FAC (0.07%) were detected in perfusates, thus demonstrating that FHPA and FAC arise from FBAL catabolism. As FAC is a well-known cardiotoxic poison, and FHPA is also cardiotoxic at high doses, the cardiotoxicity of FU might stem from at least two sources. The first one, established in previous papers (Lemaire et al, 1992, 1994), is the presence in commercial solutions of FU of degradation products of FU that are metabolized into FHPA and FAC; these are formed over time in the basic medium necessary to dissolve the drug. The second, demonstrated in the present study, is the metabolism of FU itself into the same compounds.


British Journal of Cancer (1998) 77(1), 79–86
© 1998 Cancer Research Campaign

### The anti-cancer drug 5-fluorouracil is metabolized by the isolated perfused rat liver and in rats into highly toxic fluoroacetate

**M Arellano[1], M Malet-Martino[1], R Martino[1] and P Gires[2]**

[1]Biomedical NMR Group, IMRCP Laboratory, Université Paul Sabatier, 118, route de Narbonne, 31062 Toulouse, France; [2]CRVA Drug Discovery Department, Rhône Poulenc-Rorer, 3, rue de la Digue d'Alfortville, 94140 Alfortville, France

**Summary** We report the first demonstration of the biotransformation of the anti-cancer drug 5-fluorouracil (FU) into two new metabolites, α-fluoro-β-hydroxypropionic acid (FHPA) and fluoroacetate (FAC), in the isolated perfused rat liver (IPRL) and in the rat in vivo. IPRL was perfused with solutions of pure FU at two doses, 15 or 45 mg $kg^{-1}$ body weight, and rats were injected i.p. with 180 mg of FU $kg^{-1}$ body weight. Fluorine-19 NMR analysis of perfusates from IPRL and rat urine showed the presence of the normal metabolites of FU and low amounts of FHPA (0.4% or 0.1% of injected FU in perfusates from IPRL treated with 15 or 45 mg of FU $kg^{-1}$ body weight, respectively; 0.08% of the injected FU in rat urine) and FAC (0.1% or 0.03% of injected FU in perfusates from IPRL treated with 15 or 45 mg of FU $kg^{-1}$ body weight, respectively; 0.003% of the injected FU in rat urine). IPRL was also perfused with a solution of α-fluoro-β-alanine (FBAL) hydrochloride at 16.6 mg $kg^{-1}$ body weight dose equivalent to 15 mg of FU $kg^{-1}$ body weight. Low amounts of FHPA (0.2% of injected FBAL) and FAC (0.07%) were detected in perfusates, thus demonstrating that FHPA and FAC arise from FBAL catabolism. As FAC is a well-known cardiotoxic poison, and FHPA is also cardiotoxic at high doses, the cardiotoxicity of FU might stem from at least two sources. The first one, established in previous papers (Lemaire et al, 1992, 1994), is the presence in commercial solutions of FU of degradation products of FU that are metabolized into FHPA and FAC; these are formed over time in the basic medium necessary to dissolve the drug. The second, demonstrated in the present study, is the metabolism of FU itself into the same compounds.

**Keywords:** 5-fluorouracil; α-fluoro-β-alanine; $^{19}F$ nuclear magnetic resonance; metabolism; fluoroacetate; α-fluoro-β-hydroxypropionic acid; isolated perfused rat liver; rat urine

5-Fluorouracil (FU) is widely used as an anti-tumour agent for treatment of solid tumours. Its chief side-effects are myelosuppression, diarrhoea, vomiting and mucositis. However, over the last decade, the number of reports of cardiotoxicity and neurotoxicity attributed to FU has rapidly increased, probably because of the use of higher doses in continuous perfusion (Moertel et al, 1964; Rezkalla et al, 1989; Moore et al, 1990; Gamelin et al, 1991; De Forni et al, 1992; Robben et al, 1993; Anand, 1994). The precise biochemical mechanism underlying these two side-effects remains unclear, although several investigators have postulated, but never demonstrated experimentally, that FU might be transformed into fluoroacetate (FAC), a highly cardiotoxic and neurotoxic poison (Koenig and Patel, 1970; Okeda et al, 1990). FAC enters the Krebs cycle and is then transformed into fluorocitrate, which inhibits the enzyme aconitase. Aconitase catalyses the conversion of citrate to isocitrate via the obligatory intermediate *cis*-aconitate. Inhibition of aconitase leads to a build-up of citrate in animal tissues (in particular heart) and serum, and the heart production of ATP is severely limited. Toxicity and death are thought to be caused by severe impairment of energy production (Pattison and Peters, 1966; Bosakowski and Levin, 1986; Keller et al, 1996).

*Received 22 October 1996*
*Revised 17 June 1997*
*Accepted 24 June 1997*

*Correspondence to:* M Malet-Martino, Laboratoire des IMRCP, Université Paul Sabatier, 118, route de Narbonne, 31062 Toulouse Cédex 4, France

Having at our disposal a powerful method for studying the metabolism of fluorinated drugs, in particular fluoropyrimidines (Malet-Martino and Martino, 1992) we have been able to demonstrate, using fluorine-19 nuclear magnetic resonance ($^{19}F$-NMR), the biotransformation of FU into two new metabolites, α-fluoro-β-hydroxypropionic acid (FHPA) and FAC, in the isolated perfused rat liver (IPRL) and in the rat in vivo. This transformation occurs via α-fluoro-β-alanine (FBAL), the main catabolite of FU.

### MATERIALS AND METHODS

### Chemicals

FU, FAC and bovine albumin (fraction V) powder (ref. A9647) were purchased from Sigma and chromium (III) acetylacetonate ($Cr(acac)_3$) from Aldrich (all from Sigma-Aldrich Chimie, 38297 Saint-Quentin Fallavier, France). FBAL hydrochloride was provided by Tokyo Kasei Chemicals, Tokyo, Japan. 5,6-Dihydro-6-hydroxy-5-fluorouracil (FUOH) was supplied by PCR, Gainesville, FL, USA. All other chemicals were reagent grade and obtained from standard commercial sources.

### Synthesis of FHPA

FUOH (6.7 mg, 45 μmol) was dissolved in 12 ml of 1 M potassium hydroxide at ambient temperature and the mixture was stirred for 1 h (Lozeron et al, 1964). Sodium borohydride (2.3 mg, 60 μmol) was then added. After 15 min, the pH of the solution was adjusted

**Figure 1** $^{19}$F-NMR spectrum of a non-concentrated perfusate from an isolated perfused rat liver treated with FU (45 mg kg$^{-1}$ body weight) for 3 h, pH = 7.6, number of scans 18 500

to ~8.5 with 1 M perchloric acid. The precipitate was centrifuged off and the supernatant freeze dried. The mass spectral and NMR ($^{1}$H, $^{19}$F, $^{13}$C) characteristics of the fluorinated compound obtained were in accordance with the structure of FHPA.

### IPRL experiments

Male Wistar rats (Iffa Credo, Lyon, France) weighing 370–460 g were used. The IPRL experiments have been described previously (Arellano et al, 1997). The experiments were carried out with solutions prepared immediately before use at two doses for FU (15 or 45 mg kg$^{-1}$ body weight) and one dose for FBAL (16.6 mg kg$^{-1}$ body weight). The dose of 45 mg of FU kg$^{-1}$ body weight was the maximum dose that was almost entirely metabolized by the IPRL in 3 h in our perfusion conditions. The dose of 15 mg of FU kg$^{-1}$ body weight corresponds to 80 mg m$^{-2}$ in humans (De Vita et al. 1993) but, as the IPRL experiment lasts 3 h in the presence of drug in recirculating mode, this dose corresponds to ~600 mg m$^{-2}$ day$^{-1}$ of FU injected to humans as a continuous i.v. infusion, which lies within the therapeutic range (500–1000 mg m$^{-2}$ day$^{-1}$ for 4–5 days in continuous i.v. perfusion). The dose of 16.6 mg of FBAL hydrochloride kg$^{-1}$ body weight is equivalent to 15 mg of FU kg$^{-1}$ body weight. After 1 h of liver equilibration, the drug was injected into the perfusate and the experiments were continued for 3 h. At the end of the experiments, an aliquot of the perfusate was immediately frozen to –80°C until $^{19}$F-NMR analysis. This medium was called non-concentrated perfusate. The remaining perfusate was freeze dried, stored at –80°C and resuspended in ~3 ml of water immediately before $^{19}$F-NMR analysis. This represented the concentrated perfusate. Lyophilization of non-concentrated perfusate induced an increase in the pH of ~0.7 pH unit (range 0.5–0.9).

### Effects of lyophilization on the behaviour of FBAL

In basic medium, bicarbonate ions react with FBAL to give *N*-carboxy-α-fluoro-β-alanine (CFBAL), the proportion of which with respect to FBAL increases with pH up to about pH 9 (Martino et al, 1987). The perfusion medium containing $HCO_3^-$, FBAL and CFBAL were observed in the non-concentrated perfusate (Figure 1). The proportion of CFBAL relative to FBAL is much higher in the concentrated perfusate (compare Figures 1 and 2) as the pH increased after lyophilization.

The lyophilization of the perfusate led to the appearance of two signals at a chemical shift (δ) = –111.1 and –110.4 p.p.m. in the $^{19}$F-NMR spectra of concentrated perfusates from FU experiments (Fig. 2) and four signals at –111.1, –111.2, –110.3 and –110.4 p.p.m. in the $^{19}$F-NMR spectra of concentrated perfusates from FBAL experiments (Figure 3). Two experiments were carried out to show that these signals corresponded to adducts of FBAL with β- and α-glucose. First, 2.5 mg of commercial racemic FBAL hydrochloride was added to a perfusate containing neither bicarbonate (to avoid significant formation of CFBAL) nor glucose. After freeze-drying and dissolution of the residue in water, the $^{19}$F-NMR spectrum of this sample exhibited a sole signal at –112.7 p.p.m. corresponding to FBAL. After addition of 30 mg of glucose, four signals appeared. Two strong signals of equal intensity at –111.1 and –111.2 p.p.m. corresponded to the two diastereomeric adducts of racemic FBAL with β-glucose (FBAL [R]-glucβ and FBAL [S]-glucβ). The two other weak signals of equal intensity at –110.3 and –110.4 p.p.m. corresponded to the two diastereomeric adducts of racemic FBAL with α-glucose (FBAL [S]-glucα and FBAL [R]-glucα). Furthermore, addition of this sample to a concentrated perfusate from IPRL treated with FU

 © Cancer Research Campaign 1998

**Figure 2** [19]F-NMR spectrum of a concentrated perfusate from an isolated perfused rat liver treated with FU (15 mg $kg^{-1}$ body weight) for 3 h, pH = 8.3, number of scans 20 750

**Figure 3** [19]F-NMR spectrum of a concentrated perfusate from an isolated perfused rat liver treated with FBAL hydrochloride (16.6 mg $kg^{-1}$ body weight) for 3 h, pH = 8.3, number of scans 20 200

**Table 1** $^{19}$F-NMR characteristics of (1) authentic standards of α-fluoro-β-hydroxypropionic acid (FHPA) and fluoroacetate (FAC) in a concentrated blank perfusate (pH = 8.4) and (2) FHPA and FAC before and after their addition to a concentrated perfusate (pH = 8.3) from an isolated perfused rat liver experiment at 15 mg of FU kg$^{-1}$ body weight

| | FHPA | | | FAC | | |
|---|---|---|---|---|---|---|
| | δ (p.p.m.)[a] | Multiplicity[b] | $J_{HF}$ (Hz)[c] | δ (p.p.m.)[a] | Multiplicity[b] | $J_{HF}$ (Hz)[c] |
| Authentic standards | −113.7 | ddd | $^2$J 50.0<br>$^3$J 30.9, 24.1 | −141.4 | t | $^2$J 48.0 |
| Before addition | −113.7 | ddd | $^2$J 50.3<br>$^3$J 29.5, 24.9 | −141.4 | t | $^2$J 49.0 |
| After addition | −113.7 | ddd | $^2$J 50.4<br>$^3$J 30.2, 24.4 | −141.4 | t | $^2$J 48.0 |

[a] $^{19}$F-NMR δ are related to external trifluoroethanoic acid (5% (w/v) aqueous solution). [b]d, Doublet; t, triplet. [c]The slight differences in the J values are due to the low digital resolution of the spectra (1.3 Hz per point).

**Table 2** Comparison of the amounts of unmetabolized drug and metabolites in perfusates of isolated perfused rat livers treated with FU at 45 ($n$ = 4) or 15 ($n$ = 5) mg kg$^{-1}$ body weight or FBAL at 16.6 mg kg$^{-1}$ body weight ($n$ = 4) for 3 h

| | Experiments with FU at 45 mg kg$^{-1}$ body weight | | Experiments with FU at 15 mg kg$^{-1}$ body weight | | Experiments with FBAL at 16.6 mg kg$^{-1}$ body weight | |
|---|---|---|---|---|---|---|
| | μmol g$^{-1}$ of liver | Percentage with respect to injected FU | μmol g$^{-1}$ of liver | Percentage with respect to injected FU | μmol g$^{-1}$ of liver | Percentage with respect to injected FBAL |
| FU | 0.36 ± 0.40 | 3.1 ± 3.7 | 0 | 0 | 0 | 0 |
| F$^-$ | 0.48 ± 0.15 | 4.0 ± 0.9 | 0.91 ± 0.32 | 21.4 ± 6.8 | 0.58 ± 0.16 | 16.1 ± 5.6 |
| −110.1 p.p.m. | 0.05 ± 0.02 | 0.4 ± 0.1 | 0[a] | 0[a] | 0 | 0 |
| FUPA | 0.17 ± 0.04 | 1.4 ± 0.2 | 0.02 ± 0.03 | 0.6 ± 0.7 | 0 | 0 |
| FBAL + CFBAL | 6.73 ± 0.87 | 56.3 ± 6.7 | 1.28 ± 0.38 | 30.3 ± 8.6 | 1.60 ± 0.57 | 43.1 ± 11.0 |
| Total catabolites | 7.43 ± 0.94 | 62.1 ± 6.0 | 2.22 ± 0.32 | 52.3 ± 5.3 | 0.58 ± 0.16 | 16.1 ± 5.6 |
| FHPA[b] | 0.014 ± 0.003 | 0.12 ± 0.02 | 0.018 ± 0.004 | 0.42 ± 0.06 | 0.006 ± 0.0008 | 0.18 ± 0.04 |
| FAC[b] | 0.004 ± 0.0002 | 0.03 ± 0.005 | 0.005 ± 0.002 | 0.11 ± 0.04 | 0.0025 ± 0.0002 | 0.07 ± 0.007 |

[a]Only observed in one experiment out of five, representing 0.02 μmol g$^{-1}$ of liver and 0.5% of injected FU. [b]FHPA and FAC could only be assayed in the concentrated perfusate.

and thus only containing metabolic FBAL in the [R] configuration led to an increase in the signals at −110.4 p.p.m. (FBAL [R]-glucα) and −111.1 p.p.m. (FBAL [R]-glucβ).

### Rat urine

Eight rats were injected i.p. with a solution of pure FU at a dose of 180 mg kg$^{-1}$ body weight. This dose corresponds to ~950 mg m$^{-2}$ in humans (De Vita et al, 1993), which lies in the upper part of therapeutic range. Urine samples were collected over 24 h after the injection in two 12-h fractions. They were immediately frozen and stored at −80°C until $^{19}$F-NMR analysis.

### NMR spectroscopy

$^{19}$F-NMR spectra were recorded at 282.4 MHz on a Bruker WB-AM 300 spectrometer in the conditions described previously (Arellano et al. 1997). The pulse interval was 1.4 s for quantification of concentrated perfusates and 3.4 s for quantification of non-concentrated perfusates and urine samples. $Cr(acac)_3$ was added to non-concentrated perfusates and urine samples. With the NMR recording conditions used, fully relaxed spectra were obtained as the intensities of the signals were not affected by recording the spectra with a much longer repetition time (10 s). Peak areas were therefore directly proportional to concentrations. The δ values were reported relative to the resonance peak of trifluoroethanoic acid (5% (w/v) aqueous solution) used as external chemical shift reference.

We determined the amounts of FU and its different already known catabolites from the values measured in the non-concentrated perfusates. $^{19}$F-NMR is not a very sensitive analytical technique. The detection threshold depends on the spectrometer magnetic field: ~5 μM with our 7-Tesla spectrometer (Malet-Martino and Martino, 1992), ~3 μM with a 9.4-Tesla spectrometer (Kamm et al, 1996), ~1–2 μM with a 11.7-Tesla spectrometer (Hull et al, 1988). As FHPA and FAC concentrations did not reach this limit in the non-concentrated perfusates (maximal concentrations <2 μM for FHPA and <1 μM for FAC as estimated from assay of concentrated perfusates), these compounds were not detectable. We therefore determined their concentrations from the spectra of the concentrated perfusates in which FHPA and FAC concentrations were ≥10 μM and thus could be accurately assayed.

### RESULTS

### IPRL experiments with FU or FBAL

#### *Qualitative analysis*

IPRL were treated with pure FU at two doses, 45 mg kg$^{-1}$ body weight ($n$ = 4) or a 'therapeutic' dose of 15 mg kg$^{-1}$ body weight ($n$ = 5) for 3 h.

© Cancer Research Campaign 1998

**Table 3** Urinary excretion of FU and metabolites in rats treated with pure FU at 180 mg $kg^{-1}$ body weight

| Compound | Fraction 0–12 h | Fraction 12–24 h |
|---|---|---|
| Unmetabolized FU | 18 ± 11 | 0.1 ± 0.1 |
| $FUH_2$ | 0.04 ± 0.03 | 0.004 ± 0.003 |
| FUPA | 1.1 ± 0.5 | 0.2 ± 0.2 |
| FBAL | 28 ± 7 | 4 ± 4 |
| $F^-$ | 4 ± 1 | 1 ± 1 |
| FHPA | 0.08 ± 0.02 | 0.03 ± 0.02 |
| FAC | 0.003 ± 0.002 | 0.001 ± 0.001 |
| Total catabolites | 33 ± 7 | 5 ± 5 |
| Total excreted | 51 ± 17 | 5 ± 5 |

**Table 4** Comparison of the performances of the current analytical techniques for FAC determination

| Method | Minimal amount of FAC detected[a] (nmol) | Minimal amount of FAC required for the entire assay[a] (nmol) |
|---|---|---|
| HPLC (Ray et al, 1981) | 0.01 | 200[b] |
| HPLC (Kramer, 1984) | 0.015 | 20[c] |
| GC (Okuno et al, 1982) | $5 \times 10^{-5}$–$10^{-4}$ | 2[d] |
| GC (Ozawa and Tsukioka, 1987) | | 0.3[c] |
| Capillary GC (Burke et al, 1989) | | 10 or 1[c,e] |
| Headspace GC (Mori et al, 1996) | | 5[c] |
| Bioassay (Wong et al, 1995) | | 25[f] |
| $^{19}$F-NMR (this study) | 10 | 10 |

[a]FAC was previously derivatized for all GC and HPLC assays. [b]From canine gastric content fortified with FAC. [c]From water fortified with FAC. [d]From coyote stomach fortified with FAC. [e]Limit of detection with flame ionization detector or selected ion monitoring-GC/MS respectively. [f]From bait materials fortified with FAC.

A characteristic $^{19}$F-NMR spectrum of a non-concentrated perfusate shows the signals of FU at δ = –93.3 p.p.m. (except in the experiments at 15 mg $kg^{-1}$ body weight, in which the drug was entirely metabolized) and its main catabolites, α-fluoro-β-ureido-propionic acid (FUPA) at –110.7 p.p.m., FBAL at –112.4 p.p.m., CFBAL derived from the interaction of bicarbonate ion with FBAL (Martino et al, 1987) at –110.9 p.p.m. and fluoride ion ($F^-$) from the defluorination of FBAL (Martino et al, 1985; Porter et al, 1995) at –43.5 p.p.m. A weak additional signal at –110.1 p.p.m. corresponding to an unknown compound was observed in the spectra of perfusates from experiments at 45 mg of FU $kg^{-1}$ body weight and in one out of the five experiments at 15 mg of FU $kg^{-1}$ body weight. 5,6-Dihydro-5-fluorouracil ($FUH_2$) was not observed in any of the experiments (Figure 1).

A characteristic $^{19}$F-NMR spectrum of a concentrated perfusate from an IPRL treated with FU (Figure 2) shows the signals of FU at –93.2 p.p.m. (except in the experiments at 15 mg $kg^{-1}$ body weight), FBAL at –112.7 p.p.m., CFBAL at –111.5 p.p.m. and $F^-$ at –49.7 p.p.m. The differences in the values of δ in non-concentrated and concentrated perfusates are mainly due to the much higher ionic strength in the concentrated perfusates and to differences in pH (7.6 vs 8.3 respectively). The strong resonance at –111.1 p.p.m. and the weak signal at –110.4 p.p.m. are artifacts of freeze-drying. These two signals correspond to adducts of metabolic FBAL in the R configuration (Gani et al, 1985) with β-glucose (FBAL [R]-glucβ, δ = –111.1 p.p.m.) and α-glucose (FBAL [R]-glucα, δ = –110.4 p.p.m.) as demonstrated in the Materials and methods section. The two signals at –113.7 p.p.m. and –141.4 p.p.m. were assigned to FHPA and FAC, respectively, and were positively identified by spiking a perfusate with authentic standards. The recording of $^1$H-coupled or -decoupled $^{19}$F-NMR spectra after addition of authentic FHPA and FAC to a concentrated perfusate from an IPRL experiment at 15 mg of FU $kg^{-1}$ body weight showed an increase in the signals located at –113.7 and –141.4 p.p.m., respectively, with the same coupling constants (Table 1).

IPRL ($n$ = 4) were treated with commercial racemic FBAL at a dose of 16.6 mg $kg^{-1}$ body weight, dose equivalent to 15 mg of FU $kg^{-1}$ body weight. A characteristic $^{19}$F-NMR spectrum of a non-concentrated perfusate shows the signals of FBAL at –112.4 p.p.m., CFBAL at –110.9 p.p.m. and $F^-$ at –43.5 p.p.m. A characteristic $^{19}$F-NMR spectrum of a concentrated perfusate (Figure 3) shows the signals of FBAL at –112.7 p.p.m., CFBAL at –111.5 p.p.m., $F^-$ at –49.7 p.p.m., the four adducts of racemic FBAL with glucose (FBAL [R]-glucβ, δ = –111.1 p.p.m.; FBAL [S]-glucβ, δ = –111.2 p.p.m.; FBAL [R]-glucα, δ = –110.4 p.p.m.; FBAL [S]-glucα, δ = –110.3 p.p.m.), FHPA at –113.7 p.p.m. and FAC at –141.4 p.p.m.

The control experiments previously described (Arellano et al, 1997) have unambiguously shown that FAC and FHPA did not arise from a chemical transformation of FU or FBAL taking place during the perfusion experiment or the freeze-drying step but were formed via a metabolic process.

### *Quantitative analysis*

We determined the amounts of unmetabolized FU (in the 45 mg of FU $kg^{-1}$ body weight experiments), its different catabolites (FUPA, CFBAL, FBAL, $F^-$) and the unknown compound at –110.1 p.p.m. from the values measured in the non-concentrated perfusates. For FBAL experiments, the amounts of unmetabolized FBAL and $F^-$ were determined in the non-concentrated perfusates. As FHPA and FAC were not detectable in the non-concentrated perfusates, we determined their concentrations from the spectra of the concentrated perfusates. It should be noted that the amounts of FHPA and FAC were underestimated as demonstrated previously (Arellano et al, 1997).

Table 2 shows the results of the IPRL experiments. All FU was metabolized at the 15 mg $kg^{-1}$ body weight dose, whereas at 45 mg $kg^{-1}$ body weight only 3 ± 4% of the injected FU was recovered unchanged in the perfusate. The amount of total catabolites (FUPA + CFBAL + FBAL + $F^-$ + the compound resonating at –110.1 p.p.m. when present) increased as a direct function of the injected FU dose. At 45 mg of FU $kg^{-1}$ body weight, FBAL was by far the main catabolite as it represented 91% of the metabolites of FU whereas $F^-$ made up only 6%. At 15 mg of FU $kg^{-1}$ body weight, FBAL and $F^-$ represented 58% and 41%, respectively, of FU metabolites. Only small amounts of FHPA and FAC were found in the perfusates: FHPA represented 0.4% or 0.1% and FAC 0.1% or 0.03% of the injected FU (15 or 45 mg of FU $kg^{-1}$ body weight respectively). There was no significant difference in the amounts for the two doses of FU (Student's $t$-test, $0.05 < P < 0.1$ for FHPA and $0.1 < P < 0.375$ for FAC).

In FBAL experiments, $F^-$ was the main metabolite and represented 16% of the injected FBAL dose. Low amounts of FHPA and FAC were also found in the perfusates. The difference between their amounts and those determined in FU 15 mg $kg^{-1}$ body weight experiments was significant (Student's *t*-test, $P < 0.0005$ for FHPA and $0.025 < P < 0.05$ for FAC).

### Rat experiments

To check that FU was also metabolized into FHPA and FAC in vivo, a solution of pure FU was injected i.p. to eight rats at a dose of 180 mg of FU $kg^{-1}$ body weight. Urine samples were collected over 24 h after the injection in two 12-h fractions and analysed by $^{19}$F-NMR. A characteristic $^{19}$F-NMR spectrum shows the signals of FU ($\delta = -93.3$ p.p.m.) and its catabolites, $FUH_2$ at −126.0 p.p.m., FUPA at −110.6 p.p.m., CFBAL at −110.8 p.p.m. (when sample pH > 7.5), FBAL at −112.3 p.p.m. and $F^-$ at −42.6 p.p.m. FAC was detected at −140.9 p.p.m. in six and in three out of the eight samples analysed for each fraction 0–12 h and 12–24 h respectively. At the natural pH of urine samples (pH 6.2–8.2), FHPA, which was observed in all samples, produced a signal ($\delta = -112.6$ p.p.m.) within the wide base of the strong FBAL signal. For true quantification of FHPA, urine samples were also analysed at pH 2.5, which shifted the FHPA signal to −116.8 p.p.m.

The daily urinary excretion of FU and its catabolites was 56% of the injected dose and ~90% of the excretion occurred during the first 12 h (Table 3). Unmetabolized FU was almost totally excreted in the 0–12 h fractions. FBAL was by far the main metabolite as it represented 84% of the excreted metabolites. $FUH_2$ made up 0.1%, FUPA 3% and $F^-$ 13% of the excreted catabolites. Only small amounts of FHPA and FAC were observed. FHPA represented ~0.1% and FAC 0.004% of the injected FU dose.

### DISCUSSION

This study demonstrates for the first time that the last catabolite of FU in IPRL and in rats is not FBAL. Metabolism progresses further giving rise to FHPA and FAC.

In order to demonstrate the biotransformation of FU into FAC, all the experiments were carried out with solutions of FU prepared immediately before use so as to avoid formation of degradation products of FU. Indeed, previous studies from our group (Lemaire et al, 1992, 1994) indicated that the cardiotoxicity of FU was due, at least in the isolated perfused rabbit heart model, to degradation compounds of this drug, namely fluoromalonic acid semi-aldehyde (FMASAld) and fluoroacetaldehyde (Facet). These are found in commercial solutions and are formed over time in the basic medium required to dissolve FU. FMASAld is chemically transformed into Facet, which is extensively metabolized into FAC. Thus, the solutions of FU injected in IPRL or in rats had to be initially devoid of these two compounds.

Mukherjee and Heidelberger (1960) failed to demonstrate the presence of FAC on paper chromatographic analysis of the urine and tissues of mice and of cat urine after injection of 6-[$^{14}$C]FU. In a study of the pharmacokinetics and tissue distribution of 3-[$^{3}$H]FBAL in rats, Zhang et al (1992), using high-performance liquid chromatography (HPLC), only detected FBAL in urine and detected mainly conjugates of FBAL with bile acids in the liver. Using $^{19}$F-NMR, Hull et al (1988) detected FHPA in the urine of patients treated with FU, although they referred to it as compound $U_2$ and it was not identified. Two explanations could account for these observations. The first is that only small amounts of FHPA and FAC are formed. The second is that chromatographic determination of FAC involves complex and specific methodological procedures (Ray et al, 1981; Okuno et al, 1982; Kramer, 1984; Ozawa and Tsukioka, 1987; Burke et al, 1989; Mori et al, 1996). The high water solubility and the high polarity of FAC make it difficult to separate from water. Moreover, water often interferes with the derivatization reaction (esterification) required to reduce polarity and improve sensitivity for GC assay or to introduce a chromophore for HPLC determination. The limits of FAC detection for currently available techniques are reported in Table 4. The minimal amount of derivatized FAC that could be detected with chromatographic techniques is much lower than the level of FAC detectable with $^{19}$F-NMR. However, the minimal amount of FAC required to carry out the entire process (extraction from aqueous medium, derivatization and sometimes column chromatography clean-up of derivatized FAC) in accurate conditions is of the same order of magnitude for $^{19}$F-NMR and chromatographic techniques (except for the GC method of Ozawa and Tsukioka, 1987). The relative lack of sensitivity of $^{19}$F-NMR is compensated by: (1) the possibility of a direct analysis of the crude sample without any extraction and/or derivatization procedures; and (2) the specific detection of fluorinated compounds avoiding the problem of interfering components often encountered in the detection of low levels of FAC (Burke et al, 1989). To our knowledge, no assay of FHPA has been reported in literature.

FBAL and $F^-$ were the main catabolites in the experiments with FU (Table 2). We have no ready explanation for the significantly higher amount of $F^-$ observed at the low dose of FU. This observation is nevertheless supported by other experiments not reported here in which commercial solutions of FU or pure FU administered in combination with cisplatin were injected into IPRL at a dose of 15 mg of FU $kg^{-1}$ body weight and led to similar data. It has recently been shown that L-alanine-glyoxylate aminotransferase II (AlaAT-II; EC 2.1.6.44) catalysed the elimination of $F^-$ from FBAL (Porter et al, 1995). The literature on the inhibitory effect of β-fluoro-α-amino acids on transaminase reactions (Walsh, 1983) indicates that the Schiff's base intermediate formed with the pyridoxal phosphate cofactor of these enzymes can eliminate HF to form an enamine that deactivates the enzyme. Moreover, a β-alanine transaminase has been isolated from *Streptomyces griseus* and was found to be fully inhibited after incubation with FBAL (Yonaha et al, 1985). It was therefore possible to envisage that the large amounts of FBAL formed in our experiments with FU at 45 mg $kg^{-1}$ body weight could inactive the defluorinating enzyme. However, Porter et al (1995) reported that AlaAT-II purified from rat liver was not inactivated significantly during 1 h of FBAL dehalogenation. To explain our data, one might therefore evoke that either the behaviour of the enzyme is different for longer periods of time (3 h in our experiments) or other(s) pyridoxal phosphate enzyme(s) is(are) involved in FBAL defluorination, as already suggested (Spears et al, 1990; Porter et al, 1995). Although we have no evidence of an identical cell penetration of the two FBAL enantiomers, the slightly lower amount of $F^-$ formed in the experiments with [RS]-FBAL relative to the experiments with FU at 15 mg $kg^{-1}$ body weight (Table 2) is in agreement with the report that [R]-FBAL was the preferred enantiomer for the defluorinating activity in rat liver homogenates (Porter et al, 1995).

The signal at −110.1 p.p.m. observed in all the FU 45 mg $kg^{-1}$ body weight experiments and in only one out of the five experiments

© Cancer Research Campaign 1998

at 15 mg of FU $kg^{-1}$ body weight, has not been identified. Its chemical shift led us to propose that it corresponds to a compound resulting from an interaction of FBAL involving its amino group with a constituent of the perfusion medium that could have been liberated by the liver. We have already detected this kind of compound in plasma containing FBAL (Martino et al, 1987). The higher level of FBAL could explain the higher level of this compound in the experiments at 45 mg of FU $kg^{-1}$ body weight.

By analogy with the metabolism of β-alanine (Griffith, 1986), we propose the following scheme for the metabolism of FBAL. The enzymes of β-alanine catabolism are probably also involved in the catabolism of FBAL. FMASAld and Facet were not detected in our experiments as they are very reactive. The spontaneous decarboxylation of malonic acid semi-aldehyde, the non-fluorinated analogue of FMASAld, is well documented (Pihl and Fritzson, 1955). During the metabolization of fluorinated ethanes into FAC in rats, intermediate Facet was also undetected in urine and kidney extracts (Keller et al, 1996).

$$\underset{\text{FBAL}}{H_2N\text{-}CH_2\text{-}CHF\text{-}COOH} \rightarrow \underset{\text{FMASAld}}{\{OHC\text{-}CHF\text{-}COOH\}} \rightarrow \underset{\text{Facet}}{\{OHC\text{-}CH_2F\}} \rightarrow \underset{\text{FAC}}{HOOC\text{-}CH_2F}$$

$$\downarrow$$

$$\underset{\text{FHPA}}{HOH_2C\text{-}CHF\text{-}COOH}$$

The first step in β-alanine catabolism is a transamination reaction to form malonic acid semi-aldehyde catalysed by hepatic transaminases, namely β-alanine-pyruvate aminotransferase (EC 2.6.1.18), β-alanine-oxoglutarate aminotransferase (EC 2.6.1.19) and D-3-aminoisobutyrate-pyruvate aminotransferase (EC 2.6.1.40) (Griffith, 1986; Tamaki et al, 1990). Even if we found no evidence of this assertion in literature, the low levels of FHPA and FAC obtained in our experiments are probably as a result of FBAL being is a poor substrate for one or several enzymes of the catabolic pathway of β-alanine. Moreover, it has been reported that FU is a competitive inhibitor of EC 2.6.1.19 and EC 2.6.1.40 with respect to β-alanine while FBAL inactivates EC 2.6.1.40 (Kaneko et al, 1992). The larger amount of injected FU in IPRL experiments at 45 mg $kg^{-1}$ body weight could thus explain that there is no relationship between the doses of injected FU and the amounts of FHPA and FAC formed. The levels of FHPA and FAC obtained in the experiments with FBAL were nearly half the amounts formed in the experiments with FU at 15 mg $kg^{-1}$ body weight (Table 2). This is probably due to the metabolization of the sole [R] enantiomer, which is the enantiomer formed during the metabolization of FU (Gani et al, 1985).

FAC is a highly cardiotoxic and neurotoxic poison (Pattison and Peters, 1966). FHPA does not generate cardiotoxic symptoms on the isolated perfused rabbit heart model at a dose of 0.09 μmol $kg^{-1}$ body weight but is highly cardiotoxic on this model at a high dose (14 μmol $kg^{-1}$ body weight) (unpublished results). The levels of FAC and FHPA found in perfusates of rat livers and in rat urine were low. However, as the patients are normally treated for several days (even weeks) with FU at the therapeutic dose of 15 mg $kg^{-1}$, and as FAC is known to accumulate in the organism (Meldrum and Bignell, 1957), a cumulative toxicity of FAC (and possibly also FHPA) could explain cardio- and/or neurotoxic effects of FU in patients. Moreover, it has been demonstrated that FBAL, the precursor of FHPA and FAC, accumulated in rats and was retained up to 8 days in various tissues, mainly liver, heart and brain (Zhang et al, 1992). FBAL may well be further metabolized in these tissues over long periods of time. These observations could account for the delayed onset of cardiotoxic or neurotoxic symptoms with respect to the beginning of treatment in patients receiving FU (Moore et al, 1990; Anand, 1994).

The results of the present study along with those of two previous ones (Lemaire et al, 1992, 1994) show that the cardiotoxicity of FU might have at least two origins. The first is the presence of fluorinated impurities in commercial solutions of FU derived from the degradation of FU in the basic medium required for its solubilization, which are metabolized into FHPA and FAC. The second is the metabolism of FU itself into these two cardiotoxic compounds. We have demonstrated the presence of FAC and FHPA in urine of patients treated with FU (Lemaire et al, 1992, 1996). As FU solutions are not pure, FAC and FHPA could arise from both the metabolization of impurities and the metabolism of FU itself. We have shown that a part of FHPA came from FU metabolism but we could not demonstrate it for FAC (Lemaire et al, 1996).

On the basis of our results, the cardiotoxicity (and possibly the neurotoxicity) of FU could be attenuated by: (1) using formulations that are made up immediately before injection to avoid degradation of FU in solution (a lyophilisate form for example); and (2) the use of an inhibitor of the catabolism of FU (e.g. ethynyluracil (Baccanari et al, 1993) to prevent formation of FBAL and its subsequent metabolization into the toxic FHPA and FAC.

### ABBREVIATIONS

FU, 5-fluorouracil; FAC, fluoroacetate; $^{19}F$-NMR, fluorine-19 nuclear magnetic resonance; FHPA, α-fluoro-β-hydroxypropionic acid; IPRL, isolated perfused rat liver; FBAL, α-fluoro-β-alanine; $Cr(acac)_3$, chromium (III) acetylacetonate; FUOH, 5,6-dihydro-6-hydroxy-5-fluorouracil; CFBAL, *N*-carboxy-α-fluoro-β-alanine; δ, chemical shift; FBAL [R]-glucβ, FBAL [S]-glucβ, FBAL [R]-glucα, FBAL [S]-glucα, adducts of α-fluoro-α-alanine with β-glucose and α-glucose; FUPA, α-fluoro-β-ureidopropionic acid; $F^-$, fluoride ion; $FUH_2$, 5,6-dihydro-5-fluorouracil; FMASAld, fluoromalonic acid semi-aldehyde; Facet, fluoroacetaldehyde; AlaAT-II, L-alanine-glyoxylate aminotransferase II

### ACKNOWLEDGEMENTS

This work was supported by grant no. 6635 from the Association pour la Recherche sur le Cancer (to RM) and by a grant from Ligue Nationale Française contre le Cancer (Section des Hautes-Pyréenées) to MA.

### REFERENCES

Anand AJ (1994) Fluorouracil cardiotoxicity. *Ann Pharmacother* **28**: 374–378

Arellano M, Malet-Martino M, Martino R and Spector T (1997) 5-Ethynyluracil (GW776): effects on the formation of the toxic catabolites of 5-fluorouracil, fluoroacetate and fluorohydroxy-propionic acid, in the isolated perfused rat liver model. *Br J Cancer* **76**: 1170–1180

Baccanari DP, Davis ST, Knick VC and Spector T (1993) 5-Ethynyluracil (776C85): a potent modulator of the pharmacokinetics and antitumor efficacy of 5-fluorouracil. *Proc Natl Acad Sci USA* **90**: 11064–11068

Bosakowski T and Levin AA (1986) Serum citrate as a peripheral indicator of fluoroacetate and fluorocitrate toxicity in rats and dogs. *Toxicol Appl Pharmacol* **85**: 428–436

Burke DG, Lew DKT and Cominos X (1989) Determination of fluoroacetate in biological matrixes as the dodecyl ester. *J Assoc Off Anal Chem* **72**: 503–507

De Forni M, Malet-Martino MC, Jaillais P, Shubinski RE, Bachaud JM, Lemaire L, Canal P, Chevreau C, Carrié D, Soulié P, Roché H, Boudjema B, Mihura J,

© Cancer Research Campaign 1998

Martino R, Bernadet P and Bugat R (1992) Cardiotoxicity of high-dose continuous infusion fluorouracil: a prospective clinical study. *J Clin Oncol* **10**: 1795–1801

De Vita VT, Hellman S and Rosenberg SA (1993) *Cancer – Principles and Practice of Oncology*. Lippincott: Philadelphia

Gamelin E, Gamelin L, Larra F, Turcant A, Alain P, Maillart P, Allain YM, Minier JF and Dubin J (1991) Toxicité cardiaque aigüe du 5-fluorouracile: corrélation pharmacocinétique. *Bull Cancer* **78**: 1147–1153

Gani D, Hitchcock PB and Young DW (1985) Stereochemistry of catabolism of the DNA base thymine and of the anti-cancer drug 5-fluorouracil. *J Chem Soc Perkin Trans* **1**: 1363–1372

Griffith OW (1986) β-amino acids: mammalian metabolism and utility as α-amino acid analogues. *Ann Rev Biochem* **55**: 855–878

Hull WE, Port RE, Herrmann R, Britsch B and Kunz W (1988) Metabolites of 5-fluorouracil in plasma and urine, as monitored by $^{19}F$ nuclear magnetic resonance spectroscopy, for patients receiving chemotherapy with or without methotrexate pretreatment. *Cancer Res* **48**: 1680–1688

Kamm YJL, Heerschap A, Rosenbusch G, Rietjens IMCM, Vervoort TJ and Wagener DJT (1996) 5-fluorouracil metabolite patterns in viable and necrotic tumor areas of murine colon carcinoma determined by $^{19}F$ NMR spectroscopy. *Magn Reson Med* **36**: 445–450

Kaneko M, Kontani Y, Kikugawa M and Tamaki N (1992) Inhibition of D-3-aminoisobutyrate-pyruvate aminotransferase by 5-fluorouracil and α-fluoro-β-alanine. *Biochim Biophys Acta* **1122**: 45–49

Keller DA, Roe DC and Lieder PH (1996) Fluoroacetate-mediated toxicity of fluorinated ethanes. *Fundament Appl Toxicol* **30**: 213–219

Koenig H and Patel A (1970) Biochemical basis for fluorouracil neurotoxicity. *Arch Neurol* **23**: 155–160

Kramer HL (1984) Liquid chromatographic determination of sodium fluoroacetate (compound 1080) in meat baits and formulations. *J Assoc Off Anal Chem* **67**: 1058–1061

Lemaire L, Malet-Martino MC, De Forni M, Martino R and Lasserre B (1992) Cardiotoxicity of commercial 5-fluorouracil vials stems from the alkaline hydrolysis of this drug. *Br J Cancer* **66**: 119–127

Lemaire L, Malet-Martino MC, Martino R, De Forni M and Lasserre B (1994) The Tris formulation of 5-fluorouracil is more cardiotoxic than the sodium salt formulations. *Oncol Rep* **1**: 173–174

Lemaire L, Arellano M, Malet-Martino M and Martino R (1996) A novel metabolite of 5-fluorouracil in humans: 2-fluoro-3-hydroxypropionic acid. *Proc Am Assoc Cancer Res* **37**: 1225

Lozeron H, Gordon M, Gabriel T, Tautz W and Duchinsky R (1964) The photochemistry of 5-fluorouracil. *Biochemistry* **3**: 1844–1850

Malet-Martino MC and Martino R (1992) Magnetic resonance spectroscopy: a powerful tool for drug metabolism studies. *Biochimie* **74**: 785–800

Martino R, Lopez A, Malet-Martino MC, Bernadou J and Armand JP (1985) Release of fluoride ion from 5′-deoxy-5-fluorouridine, an antineoplastic fluoropyrimidine, in humans. *Drug Metab Dispos* **13**: 116–118

Martino R, Malet-Martino MC, Vialaneix C, Lopez A and Bon M (1987) $^{19}F$ NMR analysis of the carbamate reaction of α-fluoro-β-alanine, the major catabolite of fluoropyrimidines. Application to FBAL carbamate determination in body fluids of patients treated with 5′-deoxy-5-fluorouridine. *Drug Metab Dispos* **15**: 897–904

Meldrum GK and Bignell JT (1957) The use of sodium fluoroacetate (compound 1080) for the control of the rabbit in Tasmania. *Aust Veter J* **33**: 186–196

Moertel CG, Reitemeier RJ, Bolton CF and Shorter RG (1964) Cerebellar ataxia associated with fluorinated pyrimidine therapy. *Cancer Chemother Rep* **41**: 15–18

Moore DH, Fowler WC and Crumpler LS (1990) 5-Fluorouracil neurotoxicity. *Gynecol. Oncol.* **36**: 152–154

Mori M, Nakajima H and Seto Y (1996) Determination of fluoroacetate in aqueous samples by headspace gas chromatography. *J Chromatogr A* **736**: 229–234

Mukherjee KL and Heidelberger C (1960) Studies on fluorinated pyrimidines. IX. The degradation of 5-fluorouracil-6-$C^{14}$. *J Biol Chem* **235**: 433–437

Okeda R, Shibutani M, Matsuo T, Kuroiwa T, Shimokawa R, and Tajima T (1990) Experimental neurotoxicity of 5-fluorouracil and its derivatives is due to poisoning by the monofluorinated organic metabolites, monofluoroacetic acid and α-fluoro-β-alanine. *Acta Neuropathol* **81**: 66–73

Okuno I, Meeker DL and Felton RR (1982) Modified gas–liquid chromatographic method for determination of compound 1080 (sodium fluoroacetate). *J Assoc Off Anal Chem* **65**: 1102–1105

Ozawa H and Tsukioka T (1987) Gas chromatographic determination of sodium monofluoroacetate in water by derivatization with dicylohexylcarbodiimide. *Anal Chem* **59**: 2914–2917

Pattison FLM and Peters RA (1966) Monofluoro aliphatic compounds. In *Handbook of Experimental Pharmacology*, Smith FA. (ed.), pp. 387–458. Springer: New York

Pihl A and Fritzon P (1955) The catabolism of $C^{14}$-labeled-β-alanine in the intact rat. *J Biol Chem* **215**: 345–351

Porter DJT, Harrington JA, Almond MR, Chestnut WG, Tanoury G and Spector T (1995) Enzymatic elimination of fluoride from α-fluoro-β-alanine. *Biochem Pharmacol* **50**: 1475–1484

Ray AC, Post LO and Reagor JC (1981) High pressure liquid chromatographic determination of sodium fluoroacetate (compound 1080) in canine gastric content. *J Assoc Off Anal Chem* **64**: 19–24

Rezkalla S, Kloner RA, Ensley J, Al-Sarraf M, Revels S, Olivenstein A, Bhasin S, Kerpel-Fronious S and Turi ZG (1989) Continuous ambulatory ECG monitoring during fluorouracil therapy: a prospective study. *J Clin Oncol* **7**: 509–514

Robben NC, Pippas AW and Moore JO (1993) The syndrome of 5-fluorouracil cardiotoxicity. An elusive cardiopathy. *Cancer* **71**: 493–509

Spears CP, Ray M, Granger S, Diasio RB, and Gustavsson BG (1990) Dual role of serine hydroxymethyltransferase in the synergy of fluorouracil and leucovorin: effects of L-serine and glycine on $H_4$PteGlu/5,10-$CH_2$-$H_4$PteGlu ratios and $H_4$PteGlu-catalyzed release of fluoride ion from α-fluoro-β-alanine. In *Chemistry and Biology of Pteridines, 1989 Proc Int Symp Pteridines Folic Acid Deriv*, Curtius HC, Ghisla S and Blau N. (eds), Vol. 9, pp. 811–816. Walter de Gruyter: Berlin

Tamaki N, Kaneko M, Mizota C, Kikugawa M and Fujimoto S (1990) Purification, characterization and inhibition of D-3-aminoisobutyrate aminotransferase from the rat liver. *Eur J Biochem* **189**: 39–45

Walsh C (1983) Fluorinated substrate analogs: routes of metabolism and selective toxicity. *Adv Enzymol* **55**: 197–289

Wong DH, Kinnear JE and Runham CF (1995) A simple rapid bioassay for compound 1080 (sodium fluoroacetate) in bait materials and soil – its technique and applications. *Wildlife Res* **22**: 561–568

Yonaha K, Suzuki K and Toyama S (1985) Streptomyces β-alanine:α-ketoglutarate aminotransferase, a novel ω-amino acid transaminase. *J Biol Chem* **260**: 3265–3268

Zhang R, Soong SJ, Liu T, Barnes E and Diasio RB (1992) Pharmacokinetics and tissue distribution of 2-fluoro-β-alanine in rats. Potential relevance to toxicity pattern of 5-fluorouracil. *Drug Metab Dispos* **20**: 113–119

© Cancer Research Campaign 1998